# Late Onset CNS Immune Reconstitution Inflammatory Syndrome in an Immunocompetent Patient

**DOI:** 10.3389/fneur.2013.00012

**Published:** 2013-02-21

**Authors:** Alejandro Hornik, Federico Rodriguez-Porcel, Shawn Wallery, Murray Flaster, John M. Lee, José Biller

**Affiliations:** ^1^Department of Neurology, Stritch School of Medicine, Loyola University ChicagoMaywood, IL, USA; ^2^Section of Neuropathology, Department of Pathology, Stritch School of Medicine, Loyola University ChicagoMaywood, IL, USA

**Keywords:** IRIS, multifocal leukoencephalopathy, AIDS, HIV, seizures

## Abstract

Immune reconstitution inflammatory syndrome (IRIS) refers to the presence of paradoxical clinical deterioration attributable to immune system recovery during highly active antiretroviral therapy (HAART). We present an immunocompetent patient with multifocal leukoencephalopathy on HAART, with central nervous system (CNS) IRIS pathology of unknown infectious etiology. CNS IRIS pathology should be suspected in patients on longstanding HAART without immune reconstitution, presenting with unexplained leukoencephalopathy.

## Clinical Case

A 48-year-old woman had three brief consecutive episodes of dizziness and lightheadedness followed by loss of consciousness, clonic movements of the left limbs, and gaze deviation to the left. Despite being drowsy, she regained consciousness between spells. A few days prior to these episodes, she was diagnosed with a right peripheral facial paralysis and received treatment with oral acyclovir and prednisone for 10 days. Past medical history was remarkable for a 15-year history of acquired immune deficiency syndrome (AIDS). At the time of such diagnosis, she presented with recurrent pneumonias, and a CD4 count of 3 cells/mm^3^. Highly active antiretroviral therapy (HAART) was immediately initiated, she had a recovery of her immune status, remaining stable since.

At the time of presentation to our hospital, she was on atazanavir, ritonavir, tenofovir, and extended release didanosine. Two weeks prior to hospital admission, the CD4 count was 513 cells/mm^3^ and the viral load <75 copies/ml. Pertinent findings on neurological examination included a right peripheral facial paralysis and left hand and foot adventitious movements. The patient received 1 mg of intravenous (IV) lorazepam and 1000 mg of IV levetiracetam with no further spells. Levetiracetam 1000 mg every 12 h remained as scheduled medication.

**Figure 1:**
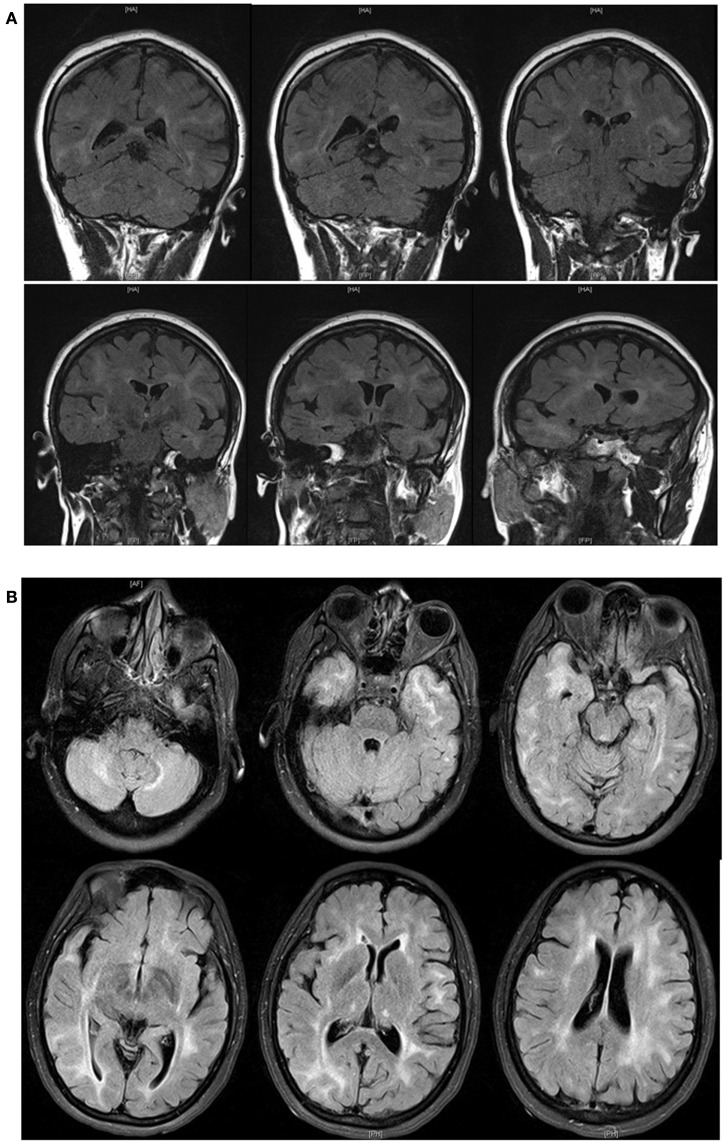
**Fluid attenuated inversion recovery (FLAIR) MRI [**(A)**,coronal; **(B)**, axial] demonstrated showing diffuse abnormalities involving the periventricular and subcortical white matter of both cerebral hemispheres, areas of punctate hyperintensities on both thalami, and foci of hyperintense signal on both cerebellar hemispheres**.

Complete metabolic profile (CMP), activated partial thromboplastin time (aPTT), international normalized ratio (INR), and prothrombin time (PT) were unremarkable. Complete blood count (CBC) showed a hemoglobin of 14.3 mg/dl and a white blood cell count (WBC) of 10.3 cells/mm^3^ (47% granulocytes, 43% lymphocytes, 8% monocytes, 1% eosinophils, and 1% basophils). Rapid plasma reagin (RPR), and *JC* polymerase chain reaction (PCR) serum testing were negative. Electroencephalogram (EEG) showed low voltage beta activity recorded diffusely with irregular polymorphic theta range activity. Focal slowing was noted intermittently from a large area of the right hemisphere. MRI of the brain with gadolinium showed diffuse T2 weighted and fluid attenuated inversion recovery (FLAIR) non-enhancing signal abnormalities involving the periventricular and subcortical white matter of both cerebral hemispheres, punctate hyperintensities on both thalami, and foci of hyperintense signal on both cerebellar hemispheres (Figure 1). Cerebrospinal fluid (CSF) analysis showed one red blood cell (RBC), seven white blood cells (WBCs; 96% lymphocytes, 11% macrophages), protein of 73 mg/dl, and a glucose of 54 (55% of serum glucose). Opening pressure was 10 cm of CSF. Cytology and flow cytometry were negative for malignant cells. CSF PCR analysis for JC virus, Herpes Simplex virus (HSV), Human Herpes virus type 6 (HHV6), Epstein Bar virus (EBV), and Varicella Zoster virus (VZV) were negative. HIV viral load on CSF was 650 copies/ml.

**Figure 2:**
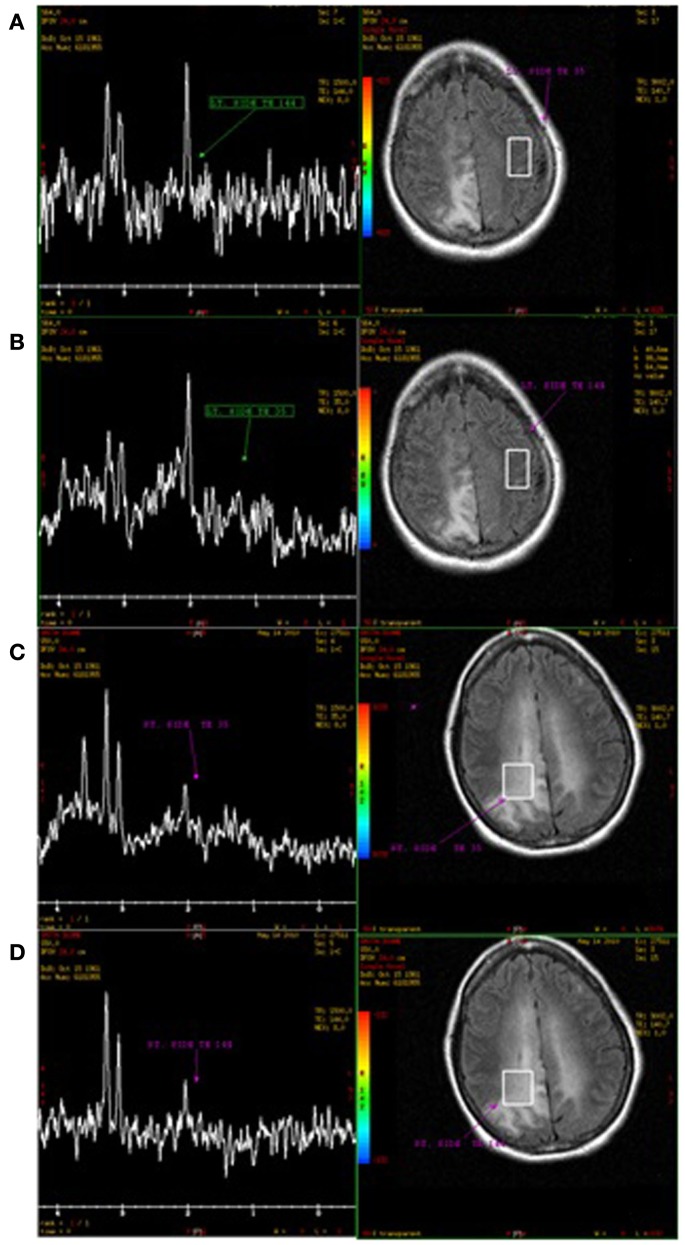
**MR spectroscopy of a right parietal lobe abnormality (C,D) demonstrate decrease in the *N*-Acetyl Aspartate peak (NAA) and elevation of the choline peak**. There is no lactate peak. While not specific, these changes could be consistent with diffuse neuronal loss and increase cellular turnover. **(A,B)** Normal parenchyma on the left parietal lobe as control.

Hospital course was remarkable for breakthrough partial complex seizures controlled with increasing doses of levetiracetam. She became forgetful and developed right-left disorientation, left sided spastic hemiparesis, and progressive gait imbalance. MR spectroscopy (MRS) of a right parietal lobe lesion was consistent with diffuse neuronal loss and increased cellular turnover (Figure 2). CT of the chest, abdomen, and pelvis was unremarkable. Bone marrow biopsy showed no hematogenous dyscrasias, normal smears, negative *EBV* PCR, and negative bacterial and fungal cultures. Serial blood cultures failed to grow any organisms. There were no signs of lymphoma affecting the eyes on ophthalmological examination.

We recommended a brain biopsy, but the patient and family declined, and only agreed to receive supportive therapy. Four weeks after presentation to our hospital, the patient became intermittently febrile and difficult to arouse. Her viral load had increased to 684 copies/ml and the CD4 count had decreased to 410 cells/mm^3^. A repeat MRI showed interval progression of the multifocal white matter lesions. A repeat CSF analysis showed 2RBCs, 23WBCs (95% lymphocytes), glucose of 58 mg/dl (52% of plasma glucose), and a protein content of 110 mg/dl. Repeat PCRs were now positive for EBV, and negative for HSV, HHV6, JC, CMV. Antibodies against Lyme disease, Cryptococcus, and Coccidioides were negative. Venereal Disease Research Laboratory (VDRL) test, cultures, smears, cytology, and flow cytometry were also negative. Serial blood cultures were negative. The patient became progressively hypotensive despite hemodynamic support with IV fluids, multiple vasopressors, and IV imipenem suffering a fatal cardiac arrest 5 weeks after initial hospital admission. A stereotatic biopsy of a right occipital lesion was performed 4 days before patient died and its final analysis was completed post-mortem.

## Pathology

**Figure 3:**
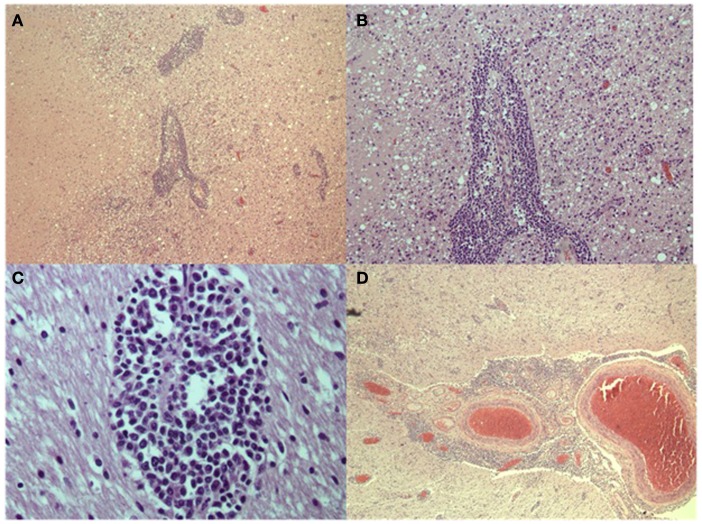
**(A,B)** (Low and medium power magnification) Primarily perivascular inflammatory infiltrates with secondary lymphocytic infiltrates in the parenchyma. **(C)** (High power) of white matter perivascular lymphocytic infiltrates. **(D)** (Low power) of perivascular and parenchymal infiltrates in the superficial cortex and leptomeninges.

**Figure 4:**
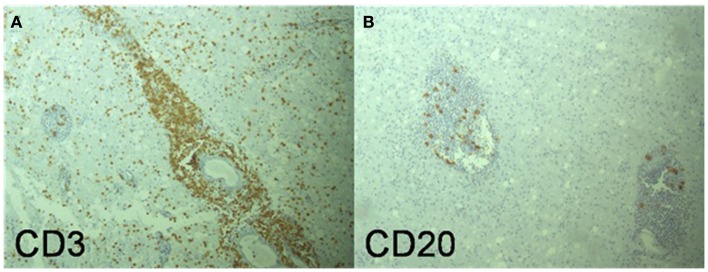
**(A)** (Medium power magnification) Primarily CD3 positive T-cell perivascular inflammatory infiltrates with secondary lymphocytic infiltrates in the brain parenchyma. **(B)** (Medium power) Some CD20 positive B-cells in perivascular area but very few in the surrounding brain parenchyma.

**Figure 5:**
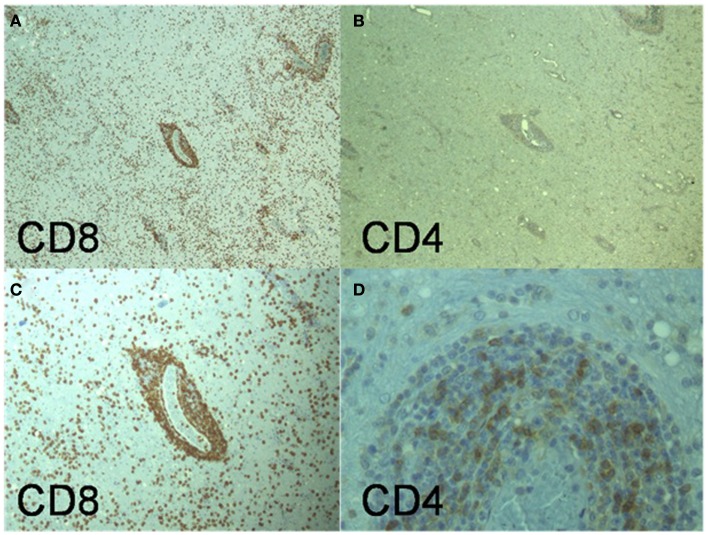
**(A,C)** (Low and medium power magnification) Primarily CD8 positive T-lymphocytes in the perivascular inflammatory infiltrates and prominent lymphocytic infiltrates in the parenchyma. **(B,D)** (Low and high power). Some CD4 positive T-lymphocytes in the perivascular areas but very few in brain parenchyma infiltrates.

At autopsy the brain showed leptomeningeal fibrosis over the convexities and moderate cerebral edema. Microscopic examination showed diffuse angiocentric and infiltrative inflammatory infiltrates throughout the brain (Figure 3). Histological features suggested a polyclonal appearance. Immunoperoxidase stains showed perivascular and parenchymal lymphocytic infiltrates primarily of CD3 positive T-cells with only some CD20 positive B-cells (Figure 4). The vast majority of the T-cells were CD8 positive with only some focal perivascular CD4 cells (Figure 5). There were also CD5 positive lymphocytes and some CD 138 and B-cell lymphoma (Bcl-1) positive cells. The infiltrates were negative for CD10, Bcl-6, and Anti-Terminal Deoxynucleotidyl Transferase (TdT). Special immunoperoxidase stains for *CMV*, *SV40* (polyoma), *HSV*, *EBV* latent membrane protein (LMP) were negative. The *EBV* *in situ* hybridization was equivocal/negative. Gram, Gomori–Grocott methenamine silver stain (GMS), AFB, and Steiner stains were negative for microorganisms. Based on the CD8+ infiltrative pattern the patient was diagnosed with Immune Reconstitution Inflammatory Syndrome (IRIS) of unknown source (Figure 3).

## Discussion

Immune reconstitution inflammatory syndrome refers to the presence of paradoxical clinical deterioration attributable to immune system recovery during HAART. IRIS can potentially involve any organ (Shelburne et al., 2002). Initial descriptions, considered IRIS in two components: the immune restoration disease (IRD), and the immune reconstitution-associated autoimmunity (Kuprica et al., 2006; French, 2009). Certain neoplasias and cellular proliferative diseases are also considered part of IRIS, exemplified by paradoxical worsening of Kaposi sarcoma after initiation of HAART (Bower et al., 2005). IRD is characterized by restoration of immunity against a specific pathogen and is further divided into an early presentation (<3 months from starting HAART) and a late presentation (>3 months after starting HAART). Early presentation IRD is best explained by an increased immune response to either subclinical or partially treated opportunistic infections. Late presentation IRD is characterized by an immune reaction induced against antigens of non-viable pathogens, and has been reported even years after onset of HAART administration (French et al., 2004; French, 2009; Martin-Blondel et al., 2011). Patients receiving HAART when markedly immunodeficient are more susceptible to IRIS (Phillips et al., 2005). A CD4+ T-cell count <100 cells/ml has been identified as a major risk factor (Phillips et al., 2005; Martin-Blondel et al., 2011). Criteria have been developed to simplify the diagnostic process (Tables 1 and 2; French et al., 2004; Shelburne et al., 2006). An HIV RNA load reduction and an increase onCD4+ T-cell count are cardinal features of this syndrome. Among patients with prior defined opportunistic infections, a meta-analysis showed a mortality rate of 4.5% and an incidence of 16.1% for all types of IRIS (Muller et al., 2010). Neuroinflammatory disease driven by IRIS has been estimated to occur in 0.9–1.5% of HIV patients started on antiretroviral agents (McCombe et al., 2009). IRIS involving the central nervous system (CNS) has been described in association with *Mycobacterial infections* (Crump et al., 1998; Foudraine et al., 1999), *Cytomegalovirus* retinitis (Jacobson et al., 1997), *JC* virus (Gray et al., 2005), *Toxoplasma Gondii*, *HSV*, *VZV*, *Parvovirus B19* (Clark et al., 2004), and *cryptococcal* meningitis (Woods et al., 1998; King et al., 2002). We could not find CNS IRIS related EBV infection. Histopathologically, IRIS is characterized by a CD8+/CD4− T-cells diffuse infiltrate, especially when produced by viruses (Mutimer et al., 2002; Gray et al., 2003; Miller et al., 2004; Rushing et al., 2008). A severe demyelinating leukoencephalopathy in AIDS patients recently started on HAART has been reported and characterized by intense astrogliosis (Langford et al., 2002; Gray et al., 2003, 2005; Miller et al., 2004). These patients usually are less immunosuppressed than the average patients with IRIS, having a CD4+ T-cell count above 200 cells/mm^3^ (Antinori et al., 2001). Although debatable whether this represents IRIS or a variant of HIV encephalopathy, we are unaware of any reports preceding the HAART era. Poor blood brain barrier penetration of most antiretroviral medications, may explain disproportionate CNS viral replication (Kandanearatchi et al., 2003). Patients, with no clear infectious etiology besides HIV have been reported to have extensive CD8+ T-cell infiltration without demyelination (Miller et al., 2004).

**Table 1:** **Working definition of IRIS (Shelburne et al., 2006)**.

a	Symptoms occurring in a patient who is HIV positive currently receiving ART
b	Immunologic Response to Antiretroviral therapy, as shown by:
	Decrease in HIV RNA levels from baseline
	Increase in CD4 cell count from baseline
c	Clinical symptoms consistent with an inflammatory process
d	Clinical course not consistent with:
	Expected course of a previously diagnosed opportunistic infection
	Expected course of a newly diagnosed opportunistic infection
	Drug toxicity

**Table 2:** **Criteria for IRIS (French et al., 2004)**.

**Diagnosis Requires Both Major Criteria or 1 Major And 2 Minor criteria**
**MAJOR CRITERIA**
Atypical presentation of opportunistic infections or tumors inpatients responding to ART: exaggerated and atypical inflammatory reaction; progressive organ dysfunction or enlargement of pre-existing lesion after definitive clinical improvement with pathogen specific therapy before starting ART; or exclusion of alternative causes (toxic effects of drug treatment, newly acquired infection or tumor or treatment failure)
**Decrease in plasma HIV RNA concentration by 1 log copies per ml**
**MINOR CRITERIA**
Increase in blood CD4 cell count after ART
Increase in an autoimmune response specific to the relevant pathogen- e.g., delayed type hypersensitivity response to mycobacterial antigens
Spontaneous resolution of disease without specific antimicrobial therapy or tumor chemotherapy with continuation of ART

Our patient presented with subacute neurological decline and multifocal leukoencephalopathy without a clear infectious etiology except for the presence of *HIV*RNA on CSF. HIV-gp41 immunoreactive staining was not performed on the autopsy specimens. The presence of one positive PCR for *EBV* in the setting of 1 negative PCR on CSF and negative studies on bone marrow and pathological specimens excluded acute *EBV* infection. Interestingly, our patient had been on HAART for over a decade with a stable CD4+ T-cell count. Unlike usual descriptions of IRIS, as the illness progressed, her CD4+ T-cell count declined and her HIV serum load increased. This paradoxical progression resembles histopathological findings of post-transplant lymphoproliferative disease (PTLD). Although PTLD usually presents as an *EBV* – B-cell disorder, it has also been described to affect T-cells (Basu et al., 2006). Miller et al. (2004) observed similar pathological findings, although their patients were significantly immunosuppressed and started on antiretrovirals 3 and 7 months prior to their clinical presentation. Patients suffering from HIV have been described to suffer a significant CD8 T-cell dysregulation characterized by multisystem infiltration (Moulignier et al., 1997). This syndrome called diffuse infiltrative lymphocytosis (DILS) manifests mainly with a Sjögren’s like syndrome and multisystem involvement. Interestingly the most common neurological manifestation of this syndrome is facial paralysis (Moulignier et al., 1997). The prevalence of DILS has significantly decreased since the advent of HAART (Basu et al., 2006). This syndrome has not been described to affect only the CNS.

## Conclusion

Our patient had a multifocal leukoencephalopathy of unknown etiology. Although autopsy was consistent with the usual pathological findings of IRIS, her clinical course and immune status were not. Our patient’s immune status remained well controlled for decades only to deteriorate as her neurological illness progressed. This is opposite to the described clinical course of IRIS were patients develop an exaggerated immune response as their immune system recovers under the effect of antiretrovirals. Our patient may have suffered from an immune dysregulation, perhaps related to intrinsic CNS HIV infection. We propose that IRIS should be diagnosed according to clinical chronological criteria using pathology only as a supplemental diagnostic tool.

## Conflict of Interest Statement

The authors declare that the research was conducted in the absence of any commercial or financial relationships that could be construed as a potential conflict of interest.
